# Barriers and enablers to breast cancer screening in rural South Africa

**DOI:** 10.4102/curationis.v47i1.2583

**Published:** 2024-09-20

**Authors:** Nelisha Sarmah, Maureen N. Sibiya, Thandokuhle E. Khoza

**Affiliations:** 1 Department of Radiography, Faculty of Health Sciences, Durban University of Technology, Durban, South Africa; 2 Division of Research, Innovation and Engagement, Mangosuthu University of Technology, Durban, South Africa

**Keywords:** breast cancer screening, rural, health education, community engagement, South Africa

## Abstract

**Background:**

The breast cancer burden on the South African healthcare system is severe, with rural South African women often diagnosed at an advanced stage of the disease. South Africa’s rural areas are classified as low-resource settings with limited medical services and infrastructure. The impact of breast cancer on rural communities in South Africa requires ongoing research to better understand the severity of this disease among one of the most vulnerable populations.

**Objectives:**

This study aimed to explore the barriers and enablers of breast cancer screening among rural South African women.

**Method:**

An exploratory qualitative study was utilised. A semi-structured interview was conducted with 22 rural South African women. Data were analysed using thematic analysis. This study utilised the care-seeking behaviour theory as its theoretical framework.

**Results:**

Participants identified many barriers to breast cancer screening, including individual affordability, transportation, rural services, infrastructure, health professional behaviour, and the lack of educational materials. Several factors are suggested to enable the screening of breast cancer in rural South Africa, including breast cancer campaigns, health education, the involvement of key stakeholders, and promotional materials.

**Conclusion:**

Despite the many barriers to breast cancer screening among rural South African women, there is still hope in implementing the various promotional tools outlined in this study and ensuring that breast self-examination is an alternative, affordable screening method.

**Contribution:**

The purpose of this article is to contribute to the attainment of the Sustainable Development Goal (SDG) 3, which focusses reducing premature mortality from non-communicable diseases, including cancer.

## Introduction

Breast cancer is the most common cancer among females in South Africa, with an incidence rate of 52.6 and a mortality rate of 16.0 per 100 000 population (Mashele et al. 2023). In black South African women, 3-year overall survival is estimated at 59.0%, mainly because of late-stage diagnosis (Ayeni et al. 2023). In previous South African studies, 63.4% of breast cancer patients of African descent were diagnosed with stages III or IV of the disease (Ansah & Vembe 2018; Ayeni et al. 2023; Kakudji et al. 2020; Warnich, Viljoen & Kuehnast 2020). Women living in rural areas in South Africa are three times more likely to be diagnosed at an advanced stage than those living in urban areas (Mapanga et al. 2023). Apartheid-era policies and ongoing socioeconomic disparities historically prevented black South African communities from accessing quality healthcare (Mashele et al. 2023). Rural South African women can experience delays in diagnosis and poorer outcomes because of a lack of access to healthcare services, including cancer screening and treatment facilities. In developed countries, mammography is the screening method of choice. However, South Africa lacks the resources to implement and sustain a national screening programme (Warnich et al. 2020). Therefore, the South African Department of Health (DoH) recommends breast self-examination (BSE) and clinical breast examination (CBE) as a means of early detection in rural communities.

Women with breast symptoms in rural South Africa are examined at primary healthcare (PHC) facilities and, if necessary, referred to secondary and tertiary healthcare facilities (Harries et al. 2020). There are, however, several barriers these women face when seeking medical care. It has been found that rural South African disparities are primarily related to factors such as education levels, unemployment, a lack of resources, poor healthcare services, and transportation. However, little research has been conducted to determine what barriers women in rural South Africa face when it comes to breast cancer screening. This article provides insight into rural South African women’s experiences and perspectives on barriers and enablers to breast cancer screening. This insight can be used to suggest tailored interventions at the individual, community, and healthcare levels.

### Problem statement

Studies conducted on women living in low-middle-income countries (LMICs) suggest that the delay between diagnosis and treatment can be influenced by a variety of factors, including knowledge and awareness, sociocultural factors, and health system barriers (Agide et al. 2019; Harries et al. 2020). The high rates of cancer mortality in South Africa are largely because of disparities in access to cancer prevention, screening, diagnosis, and treatment (Finestone & Wishnia 2022). It is commonly recognised that women from socioeconomically disadvantaged communities are faced with numerous complex medical and social conditions that, when combined, adversely impact the quality of life and the survival outcomes of women with breast cancer (Ayeni et al. 2023). South African women often present to the hospital with advanced cancer, and this occurs especially among black women (Ansah & Vembe 2018; Ayeni et al. 2023). The cancer related health seeking behaviours of South African women are influenced by cultural beliefs such as witchcraft, mistrust of medical services and the risk factors of cancer, such as smoking and alcoholism. There is a racial variation in the ages at which South African women present with breast cancer. Mixed race, Indian and black women are diagnosed at a younger age than their white counterparts. However, black women report to the hospital at advanced stages of the disease (Ansah & Vembe 2018). As such, early detection of breast cancer is essential. To better promote breast health education, a greater understanding of barriers to breast cancer screening among rural South African women is required. There is, however, little information available about the facilitators of breast cancer screening in rural South Africa. This may result in less effective health education and promotional campaigns aimed at this group of women. To ensure a tailored approach during interventional programmes aimed at rural South African women, the researchers felt it necessary to explore the barriers and enablers of breast cancer screening.

### Aim

This study aimed to explore the barriers and enablers of breast cancer screening among rural South African women.

### Theoretical framework

The study employed the Care-Seeking Behaviour (CSB) Theory as it’s theoretical framework. Lauver developed the CSB Theory based on Triandis’ Theory of Interpersonal Behaviour (Dsouza et al. 2022). In Lauver’s view, health behaviour is affected by psychosocial factors, external conditions, clinical characteristics, and sociodemographic characteristics (Orshak & Lauver 2023). Previously, the CSB Theory was applied to examine the uptake of cervical cancer screening among South Indian women (Dsouza et al. 2022). It was suggested that factors facilitating cancer screening in LMICs should be identified to ensure that more women are diagnosed at an early stage (Dsouza et al. 2022). In a previous study, health behavioural theories were examined and their application to women’s participation in mammography screenings was explored. The authors of the study found that affordability, geographic accessibility, previous healthcare experience, and acceptance of mammography screening programmes within a community are facilitators of breast cancer screening in countries with high levels of poverty (Lawal et al. 2017).

External conditions of the CSB Theory refer to those specific, objective factors relevant to care-seeking, such as affordability, accessibility, and acceptability (Orshak & Lauver 2023). The affordability of breast cancer treatment may influence a woman’s decision to seek treatment. Breast cancer screening is strongly affected by accessibility to healthcare, such as distance from a healthcare facility. Women’s willingness to seek care is believed to be influenced by the acceptability of care, such as their perception of the quality of service provided. The World Health Survey indicates that access to healthcare is a significant barrier for women in LMICs (Dsouza et al. 2022). It has been identified that education, unemployment, geographical location, and transportation are the most common barriers to breast cancer screening. Although these factors are common in most impoverished African countries and regions, they cannot be generalised. This is because of the ongoing efforts of governments in various countries to improve the current conditions of disadvantaged communities within their jurisdictions. Thus, this article will utilise the external conditions of the CSB Theory to examine the factors that facilitate breast cancer screening among rural South African women.

## Research methods and design

### Research design

A qualitative, exploratory research design was used to explore the barriers and enablers of breast cancer screening in rural South Africa.

### Setting

In this study, the setting refers to community clinics that provide PHC services. The study was conducted in four clinics within the iLembe District Municipality in KwaZulu-Natal (KZN) province. The researchers chose these clinics because they consistently serve a majority of the sample population needed for the study, compared to local churches or community centres. Each clinic is located in one of the four municipalities in the iLembe District, namely Mandeni, KwaDukuza, Ndwedwe, and Maphumulo. The district of iLembe is primarily rural and has a variety of clinics located throughout. This district has a female population of 52% (iLembe District Municipality 2021). In addition, most of these clinics are PHC facilities that provide only a limited range of services. Therefore, patients who require further diagnostic investigation and treatment are referred to tertiary health facilities located a considerable distance from these rural clinics.

### Population

In this study, the population consisted of all rural South African women over the age of 20 living in the iLembe District Municipality.

### Sampling and sampling technique

In this study, participants were selected through a purposive sampling technique. A criterion for participation was that participants be South Africans of African descent, aged 20–60 years, living in rural areas of the iLembe district in the KZN province and having consented to participate in the study. Data saturation was used to determine the sample size (Mwita 2022). In total, 22 rural South African women participated in the study.

### Data collection

The data were collected between August 2022 and October 2022. The data were obtained through semi-structured interviews using open-ended questions and probing when necessary. In this way, the participants were allowed to express themselves and provide detailed information without being restricted by closed-ended questions. To ensure confidentiality, interviews were conducted in a designated area within the clinic. The interviews were conducted in English; however, a translator was available for participants to speak in isiZulu, if they so desired. During the interviews, 21 participants did not require the assistance of a translator. However, one participant requested a translator to clarify a probing question. The translator services were therefore, utilised by only one participant. The duration of each interview ranged between 30 min and 40 min. In total, 22 interviews were conducted. Saturation of data was achieved with 18 participants, but 4 additional interviews were conducted to ensure that data saturation was reached. With the permission of each participant, the interviews were audio recorded and later transcribed verbatim.

In semi-structured interviews, several main questions are formulated to define the areas to be explored. However, they also offer a direction for the researcher or respondent to deviate to acquire feedback in more depth. There were two sections in the interview guide: Section 1, which contained the demographic information of the participants and Section 2, which contained open-ended questions, guided by the theoretical framework. The following are the main questions used:

- What is your understanding of the words ‘breast cancer’ and ‘breast self-examination’?
- What are the problems you face when you need medical care?
- In your opinion, how do you think we can encourage women in your community to practice BSE?
- If there is a programme in the community to teach women about breast cancer and BSE, will you attend? Explain.
- Does the clinic you go to give you any information on breast cancer and BSE? Explain.

Probes were used to clarify responses and to elicit more detailed information when necessary.

### Data analysis technique

Transcripts of the interviews were transcribed verbatim by the researcher, with participants’ names replaced with codes to ensure confidentiality. Thematic analysis was selected as the method of data analysis. During thematic analysis, patterns (themes) were identified, analysed, and reported within the data (Guest, Macqueen & Namey 2012). Its purpose was to provide an in-depth overview and description of the data comprehensively and interpret various aspects of the research topic (Rosairo 2023). Data from the interviews were analysed using thematic analysis.

### Trustworthiness

As recommended by Lincoln and Guba, this study was evaluated based on four criteria for establishing the trustworthiness of qualitative studies: credibility, dependability, confirmability, and transferability (Enworo 2023; Lincoln & Guba 1985). The extensive engagement with participants resulted in a high level of credibility. To ensure reliability, a record of raw data was kept for each interview. It was necessary to establish confirmability by providing an audit trail describing how data were collected, analysed, and interpreted. Through a detailed description of both the research setting and methods, the transferability of this study was assured, thereby confirming the validity and authenticity of the research. As a result, future research could be based on the findings of this study.

### Ethical consideration

Ethical clearance was obtained from the Institutional Research Ethics Committee of Durban University of Technology. In addition, permission was sought from the KZN DoH and the iLembe District Manager. Permission was granted and the reference number is KZ_202207_036. A consent form was signed by each participant before the recording of all interviews. Anonymity and confidentiality were maintained throughout all responses. Participants’ names were not used during the interview, but a number was assigned to each participant based on the order in which they were interviewed. P1 was the first person interviewed, followed by P2 up to P22. The data were stored electronically and protected by a password.

## Results

Table 1 summarises the demographics of the participants.

**TABLE 1:** Demographics of participants.

Participants	Age (years)	Level of education	Marital status	Employment status	Number of children	Municipal residence
P1	31–40	H/S	M	FT	None	Ndwedwe
P2	20–30	H/S	S	U/E	1	Ndwedwe
P3	20–30	H/S	S	U/E	3	Ndwedwe
P4	31–40	H/S	S	U/E	3	Ndwedwe
P5	20–30	T/E	S	PT	None	Ndwedwe
P6	31–40	T/E	M	FT	More than 3	Ndwedwe
P7	20–30	H/S	S	U/E	None	Ndwedwe
P8	20–30	T/E	S	FT	1	KwaDukuza
P9	41–50	T/E	W	FT	3	KwaDukuza
P10	31–40	H/S	S	PT	More than 3	KwaDukuza
P11	31–40	H/S	S	FT	1	KwaDukuza
P12	41–50	H/S	S	U/E	None	KwaDukuza
P13	51–65	T/E	S	FT	2	KwaDukuza
P14	41–50	H/S	M	FT	2	Maphumulo
P15	20–30	T/E	S	FT	2	Maphumulo
P16	31–40	H/S	S	PT	2	Maphumulo
P17	20–30	H/S	S	FT	None	Maphumulo
P18	41–50	H/S	S	PT	2	Mandeni
P19	41–50	H/S	M	U/E	More than 3	Mandeni
P20	41–50	H/S	S	U/E	1	Mandeni
P21	31–40	H/S	S	FT	2	Mandeni
P22	20–30	H/S	S	FT	2	Mandeni

*Source:* Sarmah, N., Sibiya, M.N. & Khoza, T.E., 2023, ‘The sociocultural influences on breast cancer screening among rural African women in South Africa’, *International Journal of Environmental Research and Public Health* 20(21), 1–12. https://doi.org/10.3390/ijerph20217005.

P, Participant; H/S, High School; T/E, Tertiary Education; S, Single; M, Married; W, Widowed; FT, Full-time Employed; PT, Part-time Employed; U/E, Unemployed.

### Themes and subthemes

The supporting extracts from the interviews revealed two main themes and several subthemes related to the CSB Theory (Table 2). Two researchers analysed the data and developed themes based on the findings. In the CSB Theory, ‘external conditions’ refer to factors influencing rural women’s screening behaviour. The study revealed that there are barriers and enablers that influence rural South African women’s breast cancer screening practices. To better understand the construct (external conditions) of the CSB Theory, participants were asked to describe the problems they face when seeking medical care, including breast cancer screening. This question generated several responses, allowing the researcher to probe further. Participants were questioned about breast health programmes, educational materials, or campaigns offered by their respective communities. This was performed to better understand the level of awareness of breast cancer screening among women living in rural South Africa. As a result, participants were asked how they could promote breast cancer screening in their communities by making recommendations. The findings of each of the two themes mentioned will be presented in the following sections.

**TABLE 2:** A summary of the themes and subthemes.

Themes	Subthemes
1. Barriers to breast cancer screening	1.1 Affordability 1.2 Accessibility 1.3 Acceptability
2. Enablers to breast cancer screening	2.1 Health education 2.2 Community engagement

*Source:* Sarmah, N., Sibiya, M.N. & Khoza, T.E., 2023, ‘The sociocultural influences on breast cancer screening among rural African women in South Africa’, *International Journal of Environmental Research and Public Health* 20(21), 1–12. https://doi.org/10.3390/ijerph20217005.

#### Theme 1: Barriers to breast cancer screening

According to this theme, affordability, accessibility, and acceptability are barriers to breast cancer screening for rural women in South Africa.

**Subtheme 1.1: Affordability:** Low-income households may have difficulty accessing healthcare, resulting in delayed treatment. As a result of financial constraints, participants indicated that they were unable to obtain medical care when they were ill:

‘The problem I have when I need medical care is money. Sometimes I’m sick, I don’t have the money.’ (P13, tertiary education, single, full-time employee)

As a result, rural South African women seek alternative treatment when they are ill because of the high cost of medication and transport to the local clinic:

‘Finance and transport are a problem sometimes, especially if it is not month end. During the month it is hard, but I try my best to get to the clinic.’ (P12, high school, single, unemployed)

‘If I’m getting sick, I buy Grandpa or Disprin because we afford those treatments.’ (P3, high school, single, unemployed)

Participants were asked what they would do if they discovered a breast lump. This was to better understand the financial implications of breast cancer screening among rural South African women. Many respondents indicated that they would consult a traditional healer or seek alternative treatment, while others indicated they would ignore the lump. This has substantial implications for breast cancer late-stage diagnosis among rural South African women:

‘I will go to a traditional healer to look for a solution.’ (P3, high school, single, unemployed)

‘If it’s something painful, I get some painkillers and use them and see if the pain goes away.’ (P2, high school, single, unemployed)

‘To be honest, I would ignore it.’ (P2, high school, single, unemployed)

It was observed by several participants that they were unaware of the importance of breast cancer screening. The reason is suggestive of the financial burden most women face when deciding to visit a healthcare facility that provides access to health advice and expertise on breast cancer screening:

‘We don’t know anything about breast exams.’ (P2, high school, single, unemployed)

**Subtheme 1.2: Accessibility:** Rural South African women face healthcare challenges because of substandard healthcare services and inadequate infrastructure. There was a wide range of dissatisfaction expressed by participants. Urban and rural areas were compared concerning resource availability and access to timely medical care. As most healthcare facilities are geographically dispersed, many rural women must wait for mobile clinics to travel to their respective areas once a month:

‘Yes, we do face problems. Urban areas are different, townships are different. But in a rural area, it’s very difficult because the clinics do not come. Okay. They’re not always there. It’s always a mobile clinic that comes once after two months. After two weeks, sorry, pardon me! After two weeks. It’s very hard. It’s not as easy as it is. It’s not as sophisticated as it is in the urban area. So, it’s very difficult, because you might get sick today but be treated on Friday. So, it’s not something that you can guarantee.’ (P2, high school, single, unemployed)

In addition, several participants expressed dissatisfaction with the exceptionally long queues and waiting times at clinics located in rural areas. As a result, healthcare facilities in rural South Africa were overburdened because of the shortage of medical personnel:

‘The people at large the thing they raised their concern about the clinic’s long queues.’ (P2, high school, single, unemployed)

‘Long queues! Clinics do not like taking their job seriously. They don’t mind sitting around and chatting while we are sitting and waiting for their help. So that’s a big challenge.’ (P2, high school, single, unemployed)

Among those living in rural areas, transportation is one of the most significant challenges. Rural areas in South Africa lack transportation facilities, resulting in many people seeking delayed healthcare services. Most rural South Africans live a considerable distance from their nearest healthcare facility, in which leads them to seek alternate treatment options (traditional remedies and non-prescription medication):

‘They don’t get to me on time because where I live it’s in a rural area so there is less transport.’ (P2, high school, single, unemployed)

**Subtheme 1.3: Acceptability:** Several participants expressed their dissatisfaction with the unprofessional conduct of healthcare workers. In describing the behaviour of healthcare workers at the health facilities they visited, many of the participants used words such as ‘rude’, ‘shouting’, and ‘embarrassing’. Because of the poor behaviour and service provided by healthcare workers, patients often fail to take their medications or fail to visit a health facility to receive treatment for a health issue or to undergo breast cancer screenings. After further exploration, the researcher discovered that two participants had experienced personal encounters with healthcare workers:

‘From my side, I have a problem! The nurses scream about which room I must go to. For example, I wanted to have an abortion and then I asked which room they do that. She said: “[*T*]he abortions are done in room number 10” and everyone started looking at me.’ (P2, high school, single, unemployed)

‘Sometimes you feel like I want to go to the clinic, but I can’t go there because they didn’t help me. They’re going to embarrass me instead of helping me. When you are HIV positive, when you default, they embarrass you. “Why did you default?” They do not ask you, your side, why you default the pills then you explain. They just embarrass you. So that’s the problem we end up not going to the clinic to take our medications.’ (P2, high school, single, unemployed)

‘Participants also expressed concern about the poor quality of services provided by allied health practitioners, namely paramedics.’ (P2, high school, single, unemployed)

‘When you call the ambulance to come, they take so long. When they come, they don’t take you. They want to take you to the hospital when you don’t stand up or walk or something. But if you call the ambulance, you are walking but you are sick, they don’t take you to the hospital.’ (P2, high school, single, unemployed)

For those participants who visited their local health facility, they expressed their disappointment at the lack of educational material regarding breast cancer screening. In addition, some participants found that breast cancer awareness generally occurs during ‘breast cancer month’; however, breast cancer screening programmes are extremely rare throughout the year:

‘From my understanding, they only give it if it’s breast cancer week. If not, there’s nothing. Yes, or maybe when a patient comes to consult concerning their breast then, that’s when they get it.’ (P2, high school, single, unemployed)

‘I’ve been to clinics because I have a lot of children and I don’t know anything about breast cancer even now.’ (P2, high school, single, unemployed)

#### Theme 2: Enablers to breast cancer screening

Participants were requested to make recommendations for encouraging more rural South African women to undergo breast cancer screening. According to the overall consensus, health education and community engagement should be prioritised.

**Subtheme 2.1: Health education:** Considering the barriers outlined in this study more needs to be performed to increase breast cancer screening among rural South African women. It was reported by the majority of participants that health education through a variety of platforms was an important enabler for breast cancer screening. Many participants believe that breast health education will encourage more women to participate in breast cancer screening. Furthermore, women with breast health problems will find it necessary to seek medical assistance at a health facility promptly:

‘To teach them! To have a meeting! Give health education. It’s the only way we can help them.’ (P2, high school, single, unemployed)

‘I think that they must come and educate the people from the community.’ (P2, high school, single, unemployed)

Because of the high prevalence of HIV in South Africa, there are several initiatives to promote health education among South Africans. The participants recommend breast cancer screenings to be combined with HIV campaigns targeting rural South African women. For a community with limited resources, this may represent a cost-effective alternative:

‘You see as people are being educated on HIV, they should also be educated more like that because with HIV everyone knows about it. Yes, they know what to do even though it is still a stigma but with breast cancer, because it’s not taken as a stigma, no one sees the importance of teaching it to people.’ (P2, high school, single, unemployed)

According to some participants, simply handing out brochures without explaining or demonstrating BSE practices is ineffective. In the light of this, some participants felt that healthcare facilities should provide health education through group talks and pamphlets. As a result, BSE will be explained and demonstrated in greater detail:

‘They can health educate us and then give us pamphlets to go read at home.’ (P2, high school, single, unemployed)

It was again highlighted that group demonstrations and talks are necessary as several rural women are not able to read or understand posters or pamphlets:

‘They just place the posters. The problem is some people are not educated in the rural areas. They can’t read English. They can’t even read isiZulu or isiXhosa because they didn’t go to school.’ (P2, high school, single, unemployed)

**Subtheme 2.2: Community engagement:** It was noticed that community engagement was one of the most important enablers of breast cancer screening. The participants recommended the need for awareness campaigns targeting women in groups in rural areas of South Africa:

‘Organising programmes or campaigns, breast cancer campaigns, get women together and educate them.’ (P2, high school, single, unemployed)

‘In my opinion especially where there are more women I think when there are gatherings in the community or maybe large family gatherings, we can ask for the slots to market the breast examination and speak more about cancer.’ (P2, high school, single, unemployed)

## Discussion

This study suggests that breast cancer screening among rural South African women is influenced by individual affordability. Thirteen studies reported on financial hardships as a barrier to starting or continuing cancer treatment as well as the negative impact that the costs of treatment had on family resources (Mwamba et al. 2023). Long distances, the lack of transport, and the cost of traveling were major barriers to accessing health facilities, particularly for individuals living in rural South African settings. This was exacerbated by the long waiting times, overcrowded spaces, and the need to travel from out of town. Because of the lack of employment and financial constraints, many rural South African women seek either alternative treatment through traditional healers, non-prescription medication, or home remedies. As a result, rural South African women may not present promptly for breast-related problems or screening. Literature indicates that the use of alternative treatments in Africa is widespread. Aside from conventional healthcare, most patients with breast cancer rely on various alternative healthcare practices such as traditional, the use of organic food supplements, and spiritual and faith healing (Salisu et al. 2021). A previous study reported that the decision to opt for these alternative ways is usually linked to the lack of trust in the healthcare system, the inability to afford hospital-based care, and family and/or peer influence (Salisu et al. 2021).

The inadequate resourced health facilities in rural South Africa are an ongoing barrier to the quality of care and timely diagnosis of breast cancer. Many participants referred to the lack of screening services among a list of other services, long queues, and the unprofessional conduct of healthcare workers as some of the challenges they face when attending a rural health clinic. Previous studies also found that patients had encountered negative experiences with healthcare workers and reported that they were rude, judgemental, non-empathetic, and did not respond to patients’ questions (Mwamba et al. 2023). This is a major barrier identified towards the promotion of breast cancer screening among South Africa’s rural healthcare facilities. Many participants also complained about the long queues at rural clinics to seek medical care. There is research that has reported that the healthcare services in Africa are appalling and inadequate. The poor referral pathways, limited services, a lack of equipment and drugs, and long waiting periods often lead to delayed diagnosis and treatment (Salisu et al. 2021). The disparity between rural and urban healthcare services in South Africa was a concern expressed by several participants. The ailing services in public healthcare facilities in South Africa cause a lack of trust, which influences health-seeking behaviour, delays in hospitals visit or clinics for medical treatment, and results in severe disease (Ngene, Khaliq & Moodley 2023).

Although the barriers identified in this study may indicate a similarity, slight improvement, or further deterioration in the rural healthcare system in South Africa, it is essential to recognise that there is still hope. The hope came from the recommendations made by participants to encourage breast cancer screening in rural South African women. A couple of recurring themes emerged during the data analysis process, namely health education and community engagement. The concept of health education encompasses communication activities designed to promote positive health and prevent or diminish ill health in individuals and groups by influencing the beliefs, attitudes, and behaviours of those in power as well as the community at large (Sarmah, Sibiya & Khoza 2023; Van Teijlingen et al. 2021). Several participants recommended utilising promotional tools and engaging the community in health education. It was suggested that rural healthcare clinics should conduct more breast cancer awareness programmes using visual and audio aids, pamphlets, group talks, and demonstrations. The results of 19 studies recommended a variety of strategies for delivering breast cancer interventions, including PowerPoint presentations, group discussions, video demonstrations, training, relevant images, cards, and brochures (Noman et al. 2021). According to previous reviews, educational interventions are more effective when they are culturally relevant, language-appropriate, cognitive, personal, and model-based (Noman et al. 2021). Given the above-mentioned current barriers, it is suggested that culturally sensitive health promotion tools will be highly effective in the promotion of breast cancer screening in rural South Africa. It is evident from the constant reference made by participants to traditional healers that it is necessary to recruit highly influential community leaders (tribal leaders, traditional leaders, school educators) to increase awareness of breast cancer screening among rural South African women. A partnership between the DoH and community leaders who are centrally located, easily accessible, and highly influential in rural South African communities will enable the department to promote breast cancer screening far and wide among geographically disadvantaged and resource-limited populations.

### Strengths and limitations

This study focusses on breast cancer screening barriers and enablers in rural South Africa. Breast cancer screening may also be affected by other factors, such as traditional healers’ and tribal leaders’ perceptions of healthcare. It is recommended that future studies examine these factors in greater detail. It is acknowledged that this was a small-scale study as it does not represent all African women in South Africa. It is our intention rather to provide a snapshot of the narratives provided by a small sample of rural South African women, reflecting their perspectives in our analysis. However, a detailed description of the research methodology provides an opportunity for the study to be replicated in other settings.

### Recommendations

This study provides a platform for the South African DoH to investigate further the barriers and enablers of breast cancer screening. To address the barriers to breast cancer screening in South Africa, further research is required into the role healthcare workers play in the late diagnosis of women living in rural areas. Promotional programmes must be developed to address the lack of resources, infrastructure, and awareness regarding breast cancer screening and to reach women living in geographically remote areas of South Africa. In addition, this study identified several enablers of breast cancer screening in rural South Africa, such as tribal leaders, traditional healers, school educators, and rural healthcare workers. Therefore, future research should include these key stakeholders in breast cancer awareness campaigns and programmes. It is recommended that future research assesses the knowledge, attitude, and awareness of breast cancer screening among key stakeholders before implementing a breast cancer model that can be used in South Africa’s rural areas.

## Conclusion

A timely diagnosis of breast cancer and the referral for further investigation and treatment are essential elements of cancer care. If these conditions are not met, patients are likely to be diagnosed at an advanced stage, receive inappropriate or ineffective treatment, and ultimately suffer poor outcomes and die. Several factors play a critical role in the timely diagnosis and treatment of breast cancer at the individual, community, and provider levels. Moreover, the late presentation of breast cancer and suboptimal treatment in rural South Africa are both influenced by the affordability, accessibility, and acceptability of medical care. This qualitative study provides unique insights into the experiences and perspectives of rural South African women, as well as opportunities for tailoring interventions at the individual, community, and healthcare levels to provide unique and granular insights into factors that contribute to delays in the treatment pathway.
